# Growth Behavior of *Listeria monocytogenes* in a Traditional Norwegian Fermented Fish Product (*Rakfisk*), and Its Inhibition through Bacteriophage Addition

**DOI:** 10.3390/foods9020119

**Published:** 2020-01-22

**Authors:** Lars Axelsson, Guro Alette Bjerke, Anette McLeod, Ingunn Berget, Askild L. Holck

**Affiliations:** 1 Nofima, Norwegian Institute of Food, Fisheries and Aquaculture Research, P.O. Box 210, NO-1431 Ås, Norway; gurbje@gmail.com (G.A.B.); anemcl@so-hf.no (A.M.); ingunn.berget@nofima.no (I.B.); askild.holck@nofima.no (A.L.H.); 2 Department of Chemistry, Biotechnology and Food Sciences, Norwegian University of Life Sciences, P.O. Box 5003, N-1432 Ås, Norway; 3 Center for Laboratory Medicine, Østfold Hospital Trust, P.O. Box 300, N-1714 Grålum, Norway

**Keywords:** fermented fish, ripening conditions, *Listeria monocytogenes*, anti-*Listeria* bacteriophage, food safety

## Abstract

*Listeria monocytogenes* may persist in food production environments and cause listeriosis. In Norway, a product of concern is the traditional and popular fermented fish product “*rakfisk*”, which is made from freshwater salmonid fish by mild-salting and brine maturation at low temperatures for several months. It is eaten without any heat treatment, and *L. monocytogenes*, therefore, poses a potential hazard. We investigated the effect of salt and temperature on the growth of *L. monocytogenes* in *rakfisk* during the 91 days of maturation. The amounts of organic acids produced during fermentation were too low to inhibit growth of *L. monocytogenes*. Temperature was clearly the most important parameter for controlling *L. monocytogenes*. At 7 °C, approximately 2 log growth was observed during the first 14 days of fermentation, and the level of *L. monocytogenes* thereafter remained constant. At 4 °C, only a little growth potential of the pathogen was recorded. We also investigated the effect of the anti-*Listeria* bacteriophage P100 on *rakfisk* with added *L. monocytogenes*. The phage was introduced to the *L. monocytogenes*-inoculated fish before fermentation, and an average of 0.9 log reduction was observed throughout the fermentation period. This is the first study of *L. monocytogenes* behavior in *rakfisk* and points to possible measures for increasing the product safety.

## 1. Introduction

*Listeria monocytogenes* is a facultatively anaerobic, Gram-positive bacterium, resistant to various harsh conditions, and found ubiquitously in the environment. *L. monocytogenes* causes listeriosis, a relatively rare foodborne infection affecting mainly elderly and immunocompromised persons [1]. The incidence of the disease is relatively low (i.e., approximately five cases annually per million people per year), but the fatality rate is rather high (approximately 20%), making *L. monocytogenes* of primary concern for public health. The bacterium is found in a range of foods including dairy products, meat and egg products, seafood, freshwater fish, vegetables, and other ready-to-eat (RTE) foods [1,2], and the main cause of listeriosis is consumption of food contaminated from sources in the food processing environments [3] or at the retail level [4,5]. Legislation regarding *L. monocytogenes* in RTE food products in different countries was summarized by Jami et al. [6]. The Food and Drug Administration (FDA) in the United States of America (USA) requires absence of the bacterium in 25-g samples of RTE seafood products [7]. The European Union (EU) has a zero tolerance for *L. monocytogenes* in infant foods and RTE foods used for medical purposes, while standard RTE foods that contain less than 100 colony-forming units (CFU)/g at the end of shelf-life are accepted [8].

Fermentation of food is an ancient technique that preserves, alters the flavor, odor, and texture, and may enrich the nutritional value [9,10,11]. Fermented fish products played an important role in the food supply globally, and still do, especially in Far- and Southeast Asia. In northern Europe, only a few traditional fermented fish products are still manufactured [12]. Norwegian fermented freshwater fish called “*rakfisk*” is regarded as a national specialty food, which is manufactured in a traditional, artisanal, and localized manner. *Rakfisk* is a seasonal product, mainly consumed from late fall and through the Christmas celebration period, and it grew in popularity in Norway as a delicatessen, currently traded at approximately 400 tons annually [12]. The product is made from salmonid freshwater fish, mainly lake trout or arctic char, the former being the clearly dominant species. The most common production procedure is based on mild salting and spontaneous brine formation where the gutted fish is dry-salted and layered (belly up), preferably under pressure, in airtight containers (plastic in modern-day production). The containers are subsequently stored at low temperatures (3–7 °C) for 3–12 months, occasionally longer. The fish is totally submerged in the salt brine during the storage period (post-filling of pre-made salt brine may be necessary). Salt concentration in the brine varies among producers, but lies within a range of 4%–7% (*w/w*). During the storage, a fermentation and maturation/ripening of the fish occurs, resulting in a characteristic taste, odor, and somewhat spreadable texture of the product, attributes that increase with the ripening time. In most cases, especially in productions where low salt (4–5%) and high temperature (6–7 °C) are used, psychrotrophic and relatively salt-tolerant lactic acid bacteria (LAB), such as *Lactobacillus sakei*, dominate the fermentation [13].

Since *rakfisk* is produced and consumed without any heat treatment step, the product is the subject of food safety concerns. Historically, botulism was of major concern due to outbreaks originating from homemade productions with inadequate control of proper salt amounts and storage temperatures [12]. Present-day concerns for producers and food safety authorities revolve mostly around *L. monocytogenes* since, in theory, growth of this pathogen is not entirely inhibited by the conditions prevailing during production and storage of *rakfisk*, i.e., salt concentrations around 5% and temperatures around 5 °C [1,2]. The Norwegian Directorate of Health recommends that vulnerable groups of people, e.g., pregnant women and people with weakened immunological status due to underlying medical conditions, should avoid *rakfisk*. As a result of a relatively extensive control program, *L. monocytogenes* is from time to time detected in *rakfisk* and occasionally causes product recalls. However, outbreaks of listeriosis, where specific batches of *rakfisk* were implicated, were not recorded until fairly recently. In the last six years, two documented outbreaks (affecting three and 12 persons, respectively) and a very recent suspected outbreak occurred [14,15,16].

There are no critical control points during the production process of *rakfisk* that guarantee the complete elimination of *L. monocytogenes* in the final product. Given the ubiquitous nature of *L. monocytogenes*, the lack of listericidal steps in the production procedure, and the ability of the organism to become established in the processing environment, it is challenging to produce *rakfisk* free of *L. monocytogenes*. Adhering strictly to good manufacturing practices (GMPs) and good hygienic practices (GHPs) to prevent recontamination events and ensuring good quality of the raw materials are measures taken by the producers to limit its prevalence. However, this does not preclude the need for additional hurdles. The lytic bacteriophage P100 specifically infects the majority of *L. monocytogenes* strains [17]. It may reduce *L. monocytogenes* on a variety of RTE foods such as sliced turkey meat, cabbage, mixed seafood [18], cooked ham [19] fresh cut fruits [20], and cheese [21]. The phage was also employed for reducing *L. monocytogenes* on cold smoked salmon [22] and raw salmon fillets [23,24]. Several reviews described the potential role of bacteriophages in food safety [1,25,26,27]. The safety and efficacy of the P100 phage for reduction of pathogens on different RTE foods were evaluated by the European Food Safety Authority (EFSA) panel on biological hazards [28], and the phage was granted GRAS (generally recognized as safe) status by the FDA in USA for use up to 10^9^ plaque-forming units/g food [29]. We recently characterized the microbiota in *rakfisk* brine of six producers during the course of normal production [13]. However, to our knowledge, there are currently no publications where the actual behavior of *L. monocytogenes* in the production of *rakfisk* is described. The purpose of the present work was to determine the effect of different ripening temperatures and salt concentrations on the growth and viability of a mix of *L. monocytogenes* strains during *rakfisk* production. The chosen salt concentrations and temperatures reflect the prevailing types of commercial *rakfisk* production [13]. We also examined the effects of the bacteriophage Listex P100 on *L. monocytogenes* in brine during the *rakfisk* ripening process and on vacuum-packed *rakfisk* fillets. Our findings are of particular significance to *rakfisk* producers and food safety authorities.

## 2. Materials and Methods

### 2.1. Bacterial Strains and Culture Conditions

*L. monocytogenes* strains used in this work are listed in Table 1. Three strains originated from fish products, including *rakfisk*, marinated salmon, and herring, one strain originated from RTE meat (2230/92) and was responsible for a food outbreak in Norway in 1992 [30], and one strain was isolated from a meat processing knife [31]. The strains were maintained at −80 °C in brain heart infusion medium (BHI; Oxoid Ltd., Basingstoke, UK) supplemented with 20% glycerol (*v/v*). Rifampicin (Rif, Sigma-Aldrich, St. Louis, MO, USA)-resistant (Rif^R^) derivatives were prepared by growing strains in liquid media containing 200 µg/mL Rif as described by Heir et al. [32]. For each experiment, the strains were cultured separately overnight in BHI broth at 30 °C and then cold adapted for 24 h at the storage temperatures applied in the following experiments. Cells were subjected to centrifugation at 10,000× *g* for 10 min and washed twice with sterile phosphate-buffered saline (PBS; 10 mM Na_2_HPO_4_, 1.8 mM KH_2_PO_4_, 137 mM NaCl, 2.7 mM KCl, pH 7.4), before the cell pellets were resuspended in PBS. A five-strain suspension of *L. monocytogenes* was prepared by mixing equal amounts of washed cells (10^9^ CFU/mL) of each strain. Appropriate dilutions were prepared in PBS.

### 2.2. Rakfisk Production

**Figure 1:**
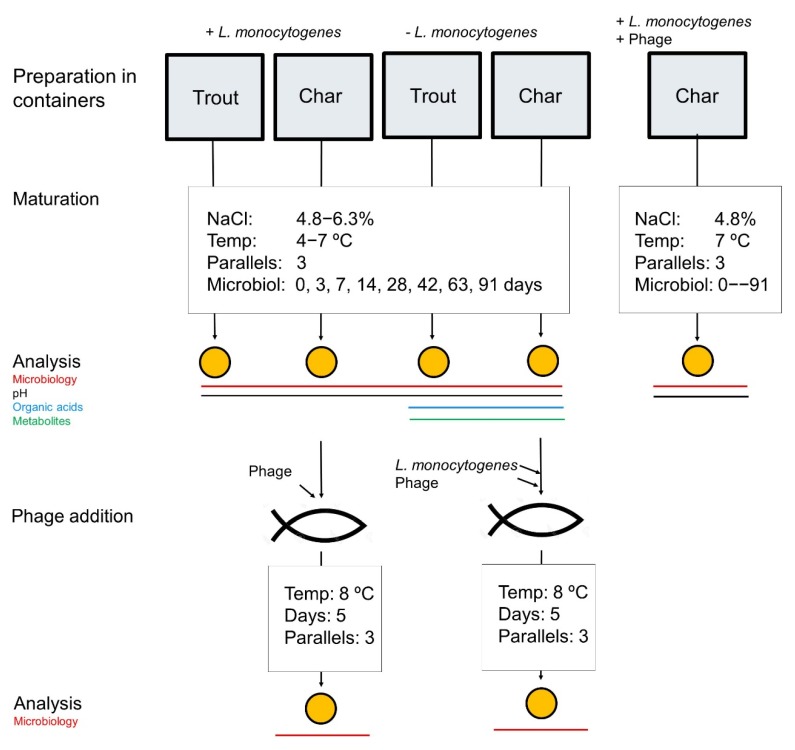
Experimental set-up and workflow.

Trout and char of approximately 600 g were obtained from Norwegian fish farmers that deliver fish to *rakfisk* producers. The fish was gutted, cleaned, and transported on ice to the laboratory facilities within 24 h after slaughtering. Fish heads, gills, and remnants of kidneys were removed before the fish was rinsed under running water and air-dried on paper. *Rakfisk* was prepared in a traditional dry salting manner [12] where fish with added salt were packed tightly in closed plastic containers (3 L), with trout and char separately. Brine was formed naturally during the first day due to the dry salting process. In cases where the fish was not completely covered with brine after 24 h, premade brine of appropriate salinity was used to fill the containers. Day zero of the experiment was set at this time-point (i.e., 24 h after packing of the containers), and the fish were submerged in brine for a ripening period of 91 days. The experimental design set-up is shown in Table 2 (C1–C16). As indicated, *rakfisk* was produced with (C1–C8) and without (C9–C16; controls) the addition of *L. monocytogenes* to the trout or char used. Production conditions were 4.8% or 6.3% NaCl (*w/w*) with storage at 4 or 7 °C. Three containers were prepared for each condition. In addition, three containers with char spiked with *L. monocytogenes* and phage P100 were prepared. The workflow and experimental set-up are schematically depicted in Figure 1.

### 2.3. Spiking of Fish with L. monocytogenes

For investigating *L. monocytogenes* viability and growth in *rakfisk* during product ripening, the fish were spiked with the *L. monocytogenes* multi-strain mix to obtain an inoculation level of 10^4^–10^5^ CFU/mL brine at day zero for easy detection of both growth and possible inactivation. The spiking procedure was as follows: 100 μL of the multi-strain mix (5 × 10^7^ CFU/mL) was added to the fish (Figure 1; Table 2, C1–C8) by dropwise distribution and spread with a bent sterile glass rod on a surface of approximately 10 cm^2^ on each side of the fish belly (muscle side). After 30 min in room temperature, the dry salting procedure was initiated by adding the appropriate amount of NaCl; finally, spiked fish were placed in the containers.

### 2.4. Rakfisk Brine Sampling

For logistic reasons explained previously and not to disturb the fermentation [13], brine samples were used for microbiological analysis. Brine samples were collected from the containers on days zero, three, seven, 14, 28, 42, 63, and 91. In order to minimize moving the fish during sampling and to avoid opening the container lids which would create a disturbance, we employed a previously described sampling system [13]. Tubes with a diameter of approximately 5 cm perforated with 1-cm^2^ holes were placed along the container side. Lids with small holes (septa sealed) aligned with the perforated tubes were used to close the containers. Brine samples from the mid layers of the containers were collected aseptically and without opening the container by using syringes with needles inserted through the septa.

### 2.5. Addition of L. monocytogenes and Phage P100 to Fish Prior to the Rakfisk Ripening Process

Char was spiked with *L. monocytogenes* as described above. After 30 min, 100 µL of P100 phage solution (PhageGuard Listex P100 phage, Micreos, Wageningen, the Netherlands), diluted to 10^10^ plaque-forming units (PFU)/mL in PBS, was spread over the same area, resulting in approximately 10^8^ PFU/cm^2^ on the fish surface. After another 30 min, the dry salting procedure was initiated by adding NaCl to a final concentration of 4.8% (*w/w*), before the fish were placed in the containers and stored at 7 °C for 91 days (Figure 1; Table 2, C17).

### 2.6. Addition of Phage P100 to Matured Rakfisk Spiked with L. monocytogenes before or after the Ripening Process

*Rakfisk* from char using 4.8% NaCl and ripened for 91 days at 7 °C, which were spiked with *L. monocytogenes* before the ripening period (Figure 1; Table 2, C6), were cut into pieces of 10 g. Phage P100 was spread onto the surface (all sides) of the pieces (2 × 100 µL per 10-g fish piece of either 10^9^ PFU/mL or 10^10^ PFU/mL) with a bent sterile glass rod, resulting in phage levels of approximately 10^8^ PFU/piece and 10^9^ PFU/piece, respectively. The pieces were then air-dried for 30 min before being vacuum-packed and stored at 8 °C for five days. Controls were fish pieces treated in the same manner, but using PBS instead of phage solution.

*Rakfisk* from char produced under the same conditions, but without added *L. monocytogenes* (Table 2, C14), were spiked with a cold adapted multi-strain mix of *L. monocytogenes*. The mix (100 µL per fish piece of 10 g at 10^7^ CFU/mL) was distributed dropwise on all sides and spread with a bent glass rod. The pieces were air-dried for 30 min. Subsequently, phage P100 was spread onto the surface as described above. After another 30 min of air-drying, the pieces were vacuum-packed and stored at 8 °C for five days. Controls were fish pieces spiked with *L. monocytogenes* in the same manner, but using PBS instead of phage solution. Both sets of vacuum-packed *rakfisk* pieces were analyzed on days one and five.

### 2.7. Enumeration of Bacteria

Enumeration of *L. monocytogenes* from fresh brine samples was carried out by plating appropriate dilutions on BHI agar (Oxoid Ltd., Basingstoke, UK) supplemented with rifampicin (200 µg/mL), incubating for four days at 30 °C. For determination of *L. monocytogenes* on fish samples, pieces of 10 g were added to 90 mL of peptone water (0.1% peptone and 0.02% Tween-80) and homogenized for 2 min in a stomacher (AESAP1064 Smasher, Cheminex, Combourg, France). Ten milliliters of the homogenate was concentrated by centrifugation at 12,000× *g* for 5 min. The supernatant containing phage P100 was removed, and the pellets containing *L. monocytogenes* cells were resuspended in 1 mL of peptone water before plating on BHI agar. Total anaerobic bacterial counts were determined by plating brine samples from one of the biological replicates collected on days zero, seven, 14, 28, 42, and 91 on plate count agar (PCA; Oxoid Ltd., Basingstoke, UK). For logistic reasons, this analysis used frozen brine samples, and all samples were processed at the same time. This procedure was previously shown not to affect the number of bacteria in *rakfisk* brine [13]. The plates were incubated anaerobically (AnaeroGen Atmosphere Generation System, Oxoid Ltd., Basingstoke, UK) for three days at 20 °C. All plating was done using an automated spiral plater (Don Whitley Scientific Limited, Shipley, UK).

### 2.8. Chemical and Metabolite Analyses

The salinity was determined in brine samples collected on day 91 by following AOAC International official volumetric method 937.09 for measuring salt (chlorine as sodium chloride) in seafood [33]. The pH was measured in brine samples using a pH meter (pH 1000 L, VWR, Leuven, Germany). For metabolite analysis, brine samples were diluted in 7.2 mM H_2_SO_4_ (1:1), and perchloric acid (PCA) precipitation was performed to remove interfering proteins. To each diluted sample (1 mL), 100 µL of PCA 35% was added. After 10 min incubation on ice, 55 µL of KOH 7 M was added to neutralize the sample and precipitate excess PCA. The samples were centrifuged at 13,000 rpm for 10 min, and supernatants were transferred to a new tube and centrifuged again for 5 min. Resulting sample supernatants were filtered through a 0.45-µm Millex-HV Durapore PVDF filter (Millipore, Burlington, MA, USA) and used for metabolite analysis. Concentrations of glucose, pyruvic acid, lactic acid, formic acid, acetic acid, acetoin, butanediol, and ethanol in the samples were determined using an Agilent 1100 series high-pressure liquid chromatography (HPLC) system (Agilent Technologies, Waldbronn, Germany) as previously described [34]. ChemStation chromatography software (Agilent) was used for data integration, and concentrations were estimated by comparison of peak areas to a calibration curve obtained with standards analyzed under the same conditions.

### 2.9. Statistical Analyses

Three containers (biological replicates) were prepared for each condition used in the *rakfisk* production experiment (Table 2), and each brine sample taken from the containers was analyzed in triplicate (technical replicates). The experiments using fish chunks (phage P100 challenge) were also performed using three biological replicates (performed on three different days) and with three technical replicates for each analysis. Statistical analyses were done after averaging technical replicates. Data are presented as averages over biological replicates with error bars corresponding to one standard error of the mean (SEM). Analysis of variance (ANOVA) was used to determine effects of fish species (Trout, Char), temperature (4 °C, 7 °C), and NaCl concentration (4.8%, 6.3%) on concentration of organic acids (lactic acid, formic acid, and acetic acid) in brine from end-point samples (day 91). Significant differences are reported when the *p*-values are below 0.05. Growth curves were analyzed using mixed linear models. The package lmerTest [35] was applied for fitting growth curves, whereas ggplot2 [36] was applied for data visualization. Growth curves for counts of *L. monocytogenes* were analyzed using mixed linear models for each fish species separately with factors temperature, NaCl concentration, day, and all two-way interactions. The full models were reduced by backward elimination keeping variables according to a significance level of α = 0.05. In addition, all treatments were compared statistically at day 14, 28, and 91 (final product) using three-way ANOVA with fish species, temperature, and NaCl concentration as factors. The growth curves for *L. monocytogenes* in the production with the addition of P100 phage were analyzed with treatment (P100 or control) and day as factors. All analyses were performed in R [37].

## 3. Results

### 3.1. Chemical Measurements

**Figure 2:**
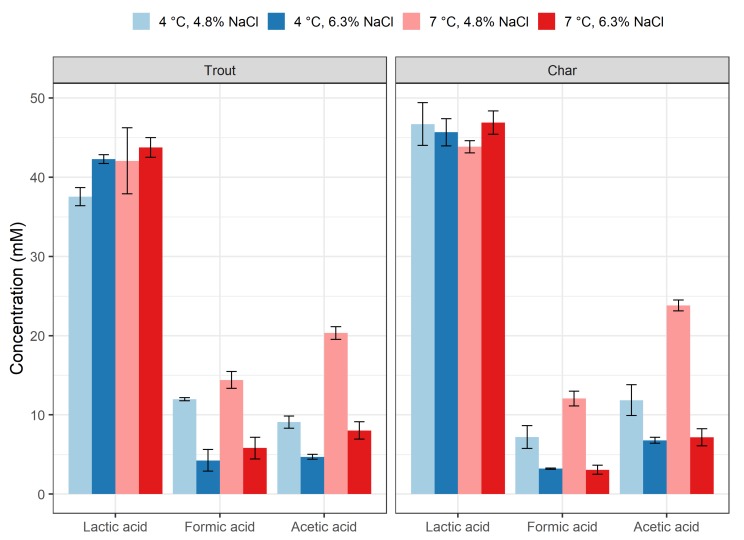
Concentrations of organic acids in *rakfisk* production at end-point (91 days). Trout and char ripened at four different combinations of conditions are shown: 4.8% NaCl and 4 °C (low salt (LS)/low temperature (LT)) indicated in light blue, 6.3% NaCl and 4 °C (high salt (HS)/LT) in dark blue, 4.8% NaCl and 7 °C (LS/high temperature (HT)) in pink, and 6.3% NaCl and 7 °C (HS/HT) in red. Vertical bars represent the standard error of the mean (SEM).

The salinity of the *rakfisk* batches were controlled at day 91 (end-point) and confirmed to be at the two intended levels of 4.8% (low salt: LS) and 6.3% (high salt; HS) (*w/w*). The pH remained essentially stable at around 6–6.5 for all conditions and time-points (not shown). Metabolites were measured in end-point (day 91) control samples (Table 2, C8–C16), of which concentrations of the organic acids most relevant for food safety (lactic, formic, and acetic acids) are displayed in Figure 2. The two fish species showed a similar pattern, with only minor differences. The lactic acid concentration was approximately 40 mM (0.36% *w/v*) in all samples with no significant differences between different temperature and salt conditions when analyzed for the same fish species. However, there was a small, but statistically significant difference in overall lactic acid concentration between the fish species (higher in char) when analyzed collectively for all conditions (Supplementary File). Significant temperature and salt effects were seen for acetic and formic acid levels, with the concentrations being higher in low-salt/high-temperature batches. For acetic acid, there was also a significant temperature–salt interaction effect which manifested itself as higher concentrations at 7 °C (high temperature; HT) compared to 4 °C (low temperature; LT) in low-salt conditions. The concentration of acetic acid in these batches reached 20 mM (0.12%) or slightly higher. Although not statistically significant, a similar trend was seen for formic acid. Glucose, pyruvic acid, acetoin, butanediol, and ethanol were not detected or were present in very small amounts.

### 3.2. Growth of L. monocytogenes in the Brine at Two Different Temperatures and Salt Concentrations during the Ripening Process of Rakfisk

**Figure 3:**
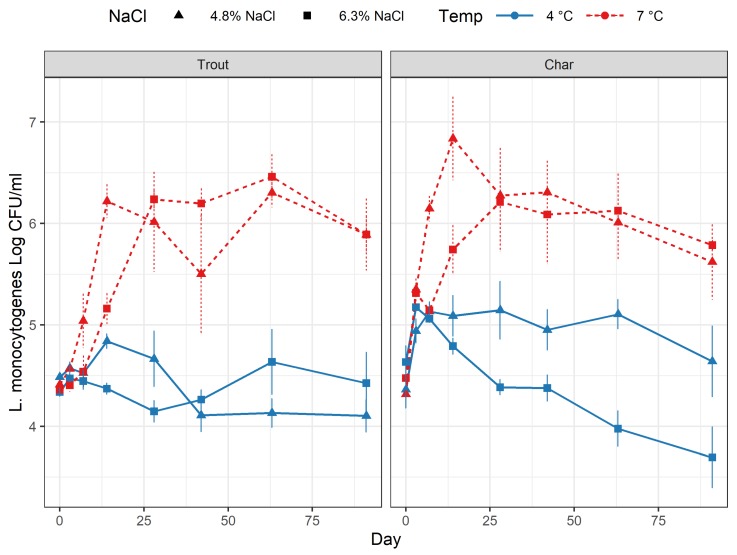
*Listeria monocytogenes* numbers in *rakfisk* brine during the ripening process for the two fish species. Left panel, trout; right panel, char. Blue line, 4 °C; red broken line, 7 °C. (■) 6.3% NaCl, (▲) 4.8% NaCl. Vertical bars represent the SEM.

The intended inoculation level of the five-strain mix of *L. monocytogenes* of 10^4^–10^5^ CFU/mL in brine at day zero was achieved with inoculation levels on the fish measured to be in the range 2.0–2.3 × 10^4^ CFU/mL. The growth and survival of *L. monocytogenes* in *rakfisk* brine of char and trout ripened at the four different combinations of conditions (Table 2) are displayed in Figure 3. Comparisons between the different curves showed that temperature was the main differentiating effect for both fish species (Supplementary File). There was also a significant temperature–day interaction effect for both trout and char, meaning that the temperature effect was different on different days. In addition, the temperature–salt–day interaction effect was significant for char, which is evident from the LT graphs (Figure 3, blue lines, right panel), showing *L. monocytogenes* numbers being significantly lower at 6.3% NaCl (HS) than at 4.8% NaCl (LS) after day 28. Results from ANOVA of the responses at specific days (day 14, 28, and 91) gave a slightly different picture, but also determined temperature as the main effect at all days. However, in the early ripening phase (day 14), the effect of salt concentration was also significant. This effect disappeared after day 28, and, at day 91, only the effect of temperature was significant. As displayed in Figure 3, the highest listerial numbers and the fastest growth were found in fish brine at 7 °C. At this temperature, *L. monocytogenes* showed rapid growth to the maximum level, which was 10^6^–10^7^ CFU/mL at day 14 for the LS batches. Growth was slightly delayed in HS batches with maximum levels reached at day 28. After this time-point, the numbers of *L. monocytogenes* remained at approximately 10^6^ CFU/mL for the rest of the ripening period, and there were no differences between LS and HS batches. For trout at 4 °C, *L. monocytogenes* numbers were essentially unchanged throughout the ripening period. For char at 4 °C, some initial growth of *L. monocytogenes* was observed early in the process. The number of *L. monocytogenes* thereafter remained essentially constant for the LS/LT samples, while a decrease was observed in the HS/LT batches, leading to the abovementioned difference in growth/survival curve appearance for this fish species.

The bacterial background flora (measured as total anaerobic counts) was only estimated in selected samples and not in all biological replicates, and a complete dataset was, therefore, not obtained. The results nevertheless indicated that bacteria were present at low levels (<100 CFU/mL) at day zero (except for batches where *L. monocytogenes* was added), and growth resulted in total numbers ranging from approximately 5 × 10^5^ CFU/mL (char, HS/LT) to 5 × 10^7^ CFU/mL (trout, LS/HT) at day 91 (Figure S1, Supplementary File). Growth appeared the fastest in the LS/HT containers with maximum numbers reached by day 28–40 and was somewhat delayed in HS and LT conditions. There were only minor differences between the fish species (Figure S1, Supplementary File).

### 3.3. Effect of Phage P100 on L. monocytogenes during the Ripening of Rakfisk

**Figure 4:**
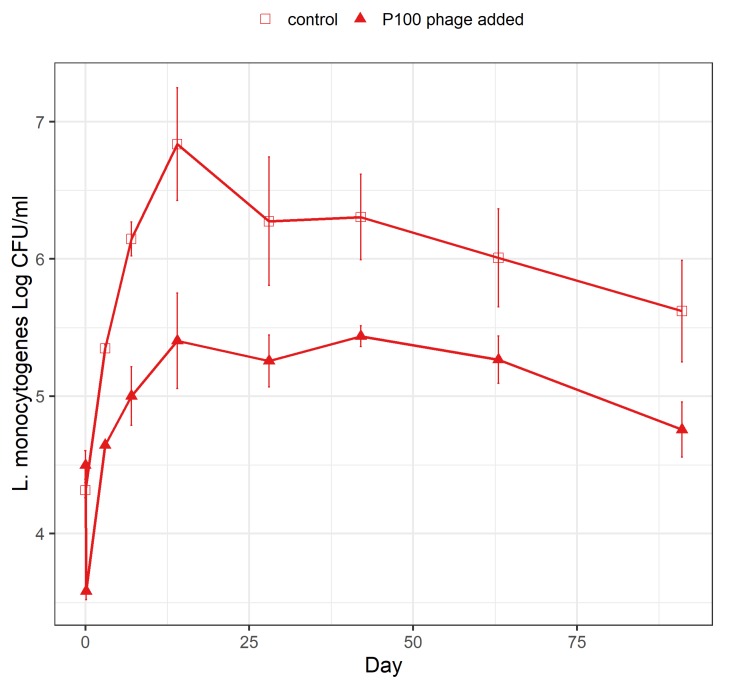
Growth and survival of *L. monocytogenes* in the presence and absence of phage P100 in char *rakfisk* brine during the ripening process at LS/HT conditions. (▲), phage P100 added at 10^8^ PFU/cm^2^ before start of the dry salting procedure; (□), control without added phage P100. Vertical bars represent the SEM.

Thirty minutes after the addition of *L. monocytogenes*, phage P100 was added to char at 10^8^ PFU/cm^2^ before the dry salting procedure and maturation (Table 2, C17). Growth and survival of *L. monocytogenes* in brine were followed during the total ripening period (91 days, Figure 4). The phage P100 addition resulted in a reduction of *L. monocytogenes* of almost 1 log CFU/mL already at the first sampling time-point (day zero) compared with fish not treated with phage P100. The reduction was maintained during the entire ripening period.

### 3.4. Effect of Phage P100 Addition on L. monocytogenes in Ripened Rakfisk Products during Short-Term Storage

**Figure 5:**
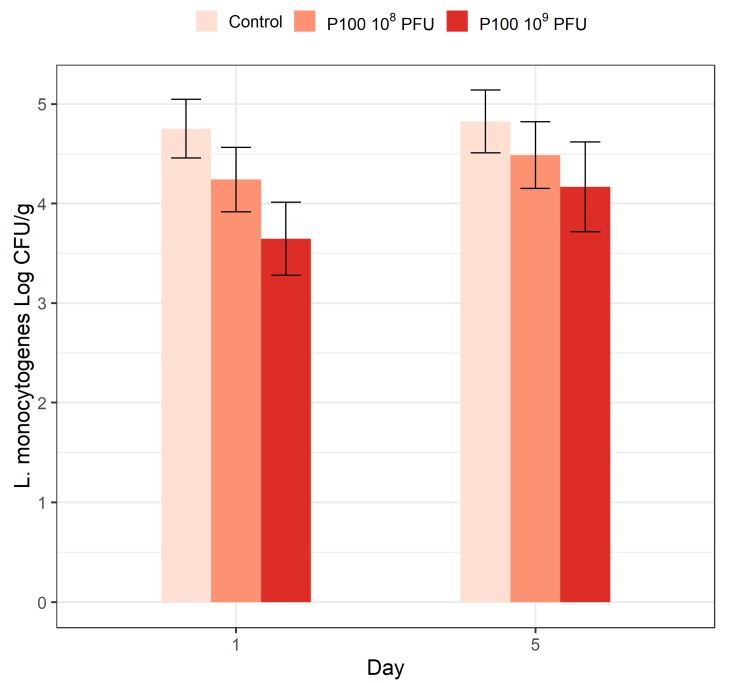
*L. monocytogenes* numbers in ripened vacuum-packed *rakfisk* in the presence of two different phage P100 concentrations. *L. monocytogenes* cells were inoculated onto the fish pieces after the ripening. Added phage P100 is indicated with different colors: brown, 10^8^ PFU/fish piece; red, 10^9^ PFU/fish piece; light brown, control without phage addition. Vertical bars represent the SEM.

Pieces of matured *rakfisk* LS/HT (Table 2, C14) were spiked with *L. monocytogenes* after the ripening period. After 30 min, the pieces were added phage P100 at two different levels before vacuum-packaging and storing at 8 °C. The levels of *L. monocytogenes* on the fish pieces, as determined by plate count, were 6 × 10^4^ CFU/g before phage addition and reduced by 0.6 and 1.2 log after one day in samples containing 10^8^ PFU/sample and 10^9^/sample PFU phage particles, respectively (Figure 5). After five days of storage, *L. monocytogenes* numbers were reduced by 0.3 and 0.6 log, respectively. There was a significant effect of phage addition with the highest dose at day one. However, the total effect of the treatment analyzed for both days and doses was not statistically significant (*p* = 0.08; Supplementary File).

In the samples where the *rakfisk* pieces originated from batches spiked with *L. monocytogenes* before the ripening process (Table 2, C6), the level of the pathogen was 3 × 10^5^ CFU/g. In this case, no effect of phage addition was found, as the level of *L. monocytogenes* after both one day and five days of storage remained unchanged (not shown).

## 4. Discussion

This work represents the first study where the behavior of *L. monocytogenes* during production of the Norwegian traditional fermented fish product, *rakfisk*, is described. The temperature and salt conditions chosen in this study essentially represent the ranges found in commercial production. The process of *rakfisk* production is generally described as a bacterial fermentation in combination with so-called “autolysis”, i.e., the activity of endogenous fish enzymes that degrade and transform proteins and fat of the fish [12,13]. Chemical analysis of end-point samples indicated that the process has lactic acid fermentation as a main component, as described previously [12], although other bacterial groups may also be prevalent [13]. Changes in temperature or salt did not change the lactic acid levels. However, the significant increase in formic and acetic acid in low-salt conditions may indicate that the microbiota composition is different in low- compared to high-salt conditions. The levels of organic acids were generally low (3–47 mM), and the pH remained essentially unchanged during the process. Previous studies showed that these levels are not inhibitory for *L. monocytogenes* at pH above 5 [38]. This was also evident in our study since the fastest growth of *L. monocytogenes* occurred in the LS/HT batches where the total concentration of acids was highest (Figure 1 and Figure 2). Low-temperature and relatively high-salt concentrations would, thus, be the major hurdles for growth of *L. monocytogenes* in standard *rakfisk* production. Our results clearly showed that temperature was the main factor influencing growth of *L. monocytogenes* in *rakfisk* brine. At 7 °C, rapid growth occurred, especially at the lower NaCl concentration (4.8%), reaching levels of 10^6^ CFU/mL well before the end of the maturation period. The inoculation level was unrealistically high in this study, but even very low contamination levels would result in numbers of food safety concern (>100 CFU/g or mL) assuming a similar growth rate. High NaCl concentration (6.3%) delayed growth somewhat, but *L. monocytogenes* eventually reached essentially the same levels also in these batches. Although even higher salt levels could inhibit the pathogen more and have a general preservative effect, this is not a realistic option, as the product will not achieve the desired *rakfisk* attributes. Low temperature (4 °C) restricted growth of *L. monocytogenes* at both salt levels. There were some variations of the numbers during the maturation period, with some initial growth in the char batches, but the level of *L. monocytogenes* at end-point (day 91) was essentially the same as at inoculation level, or somewhat lower. The variations seen in the early phase could be an effect of the salt equilibration in the dry salting and spontaneous brining process. Earlier, preliminary studies indicated that complete salt equilibration (equal concentration in fish and brine) may take 10–12 days [39].

Using a temperature of 4 °C during the maturation period, thus, seems like a feasible option for achieving some control of *L. monocytogenes* growth. However, this low temperature poses the disadvantage of prolonged *rakfisk* maturation time, and producers using this temperature generally employ a maturation time of minimum five months. In accordance, a preliminary sensory analysis of the products in our study (without *Listeria* present; Table 2, C9–C16), also indicated that only the 7 °C batches appeared ready as salable *rakfisk*. In this study we analyzed growth of *L. monocytogenes* in brine samples. It is arguable whether this is representative or not for *L. monocytogenes* contamination of the fish itself, which is the product that is consumed. However, brine samples of *rakfisk* are considered as appropriate for process control as recommended by the food safety authorities. This was corroborated by *Listeria* numbers found in both brine and fish from the source production in the severe outbreak in 2018 [14]. In addition, our results show that the batch spiked with *L. monocytogenes* before ripening and subsequently used in the phage P100 challenge experiment (see below) contained 3 × 10^5^ CFU/g fish and 6 × 10^5^ CFU/mL brine. Some samples of *rakfisk* were stored for eight and nine months. In these samples, the levels of *L. monocytogenes* remained essentially equal to those after three months of storage (not shown).

As low temperature and high NaCl concentration only lead to a limited extent of reduction of *L. monocytogenes* during production, additional strategies to reduce *Listeria* should improve the product safety. One such strategy is treatment with the bacteriophage Listex P100. In our study, the low-salt and high-temperature (LS/HT) condition was chosen as representing a “worst-case scenario”, where growth of *L. monocytogenes* was anticipated to be most prominent. The present recorded effects of phage P100 against *L. monocytogenes* in brine during the *rakfisk* ripening process and on vacuum-packed *rakfisk* fillets demonstrate their anti-listerial potential when introduced at certain steps in a production line. The contamination experiments with *L. monocytogenes* mimic likely scenarios in commercial production where fish are contaminated from the production environment or production equipment. From the salmon industry, it was documented that this route of contamination is very common [40,41]. The phage P100 treatment gave almost 1 log reduction during the fermentation and maturation period, where *L. monocytogenes* ceased to grow around day 14. The ceased growth is probably due to inhibition from the competitive background flora, which reached high levels by this day. The inhibition agrees well with that obtained using the FSSP mathematical model [42]. Under the prevailing conditions, the model suggests little growth of *L. monocytogenes* at 4 °C and about 2 log growth at 7 °C after 14 days, with no further growth thereafter when competition from lactic acid bacteria is taken into consideration (not shown). Inhibition of *L. monocytogenes* by a competitive flora was shown for other products like cooked ham [19,43]. Reports from the salmon industry describe generally low contamination levels <10 CFU/g [40], although with high prevalence. In our experiments, we used a fairly high inoculum, which would enable detection of both several log bacterial reduction and growth. For a low contamination (1–10 CFU/g), a 1 log reduction by P100 phages would lead to low final contamination levels and, therefore, to significantly reduced hazard. Thus, phage introduction against *L. monocytogenes* at the start of the *rakfisk* ripening presented an additional product safety measure. Furthermore, in the contamination experiments with *L. monocytogenes* after fermentation during packaging of the product, which also mimic a likely scenario in the production line, a weak, short-term P100 phage effect was also demonstrated. It should be noted that the number of phage per cm^2^ was relatively low in this experiment (4 × 10^7^ PFU/cm^2^ at the highest dose). Further experiments using higher doses are, therefore, warranted.

No effect of phage addition on ripened fish could be seen against *L. monocytogenes* added before fermentation, which indicates that these bacterial cells resided on the fish for an extended time and were not readily available for phage binding. The cells may be shielded in inaccessible crevices on the surface or in the complex food matrices of the fish [18] or protected in a biofilm. Again, the low number of phages/cm^2^ (see above) could have affected the outcome of this experiment.

Bacteriophages have a number of attributes that make them candidates for control of foodborne pathogens such as *L. monocytogenes*, including their self-perpetuating nature and their ability to target their host bacterium with high specificity [44]. The lytic bacteriophage P100 is a broad-host-range phage killing more than 95% of 250 different *L. monocytogenes* isolates of serovar groups 1/2 and 4. The phage genome was sequenced and no virulence genes were found [17]. From a commercial viewpoint, phage treatment constitutes a mild additional hurdle not influencing the fermentation flora of the product and not influencing organoleptic properties.

Application of phages can be carried out by simple spraying or dipping of the gutted fish before fermentation. In addition, phages may also be added after fermentation before packaging of the final product. Phages may, however, to some extent be inactivated after being sprayed on products, which varies with different foods. More importantly, intact phage particles appear to be immobilized soon after addition to solid foods and become inactive due to limited diffusion. The concentration of phages, therefore, needs to be high enough to ensure contact between phages and the target cells. Although the use of P100 phages is considered safe by EFSA, there is little legislation in Europe and Asia to regulate the use of phages in food and food production. More information is still required on immediate and long-term efficacy of phages and application methods. Potential problems are the possible emergence of phage-resistant mutants, phage spread, and inhibition in microbiological monitoring [45,46,47].

In addition to phage treatment, other measures, such as protective cultures or bacteriocins for inhibiting *L. monocytogenes*, could be relevant in *rakfisk* production.

## 5. Conclusions

The ripening temperature had the largest impact on *L. monocytogenes* growth during *rakfisk* production. Low ripening temperatures result in essentially no growth, although the conditions are not entirely inhibitory. Introducing listericidal steps such as addition of suitable bacteriophages may reduce *L. monocytogenes* numbers during the ripening process and on packed products, and this presents a possible measure for increasing the product safety.

## Figures and Tables

**Table 1:** *Listeria monocytogenes* strains used in the present work. RTE—ready to eat.

Strain No. *	Source	Reference **
MF2132	Sherry-marinated herring	NVH762
MF3508	RTE meat, *L. monocytogenes* Outbreak in Norway in 1992	Strain 2230/92 [30]
MF3509	Knife in meat factory	Strain 167 [31]
MF3511	*Rakfisk*	NVH1102
MF3512	“Gravet” (marinated) salmon	NVH1185

* Rifampicin-resistant (Rif^R^) derivatives. ** NVH strains from the Norwegian University of Life Sciences, Oslo, Norway.

**Table 2:** Experimental design of the *rakfisk* production experiments.

Condition ^a^	Fish Species	*L. monocytogenes* Added	NaCl (% *w/w*)	Temperature (°C)
1	Trout	+	4.8	4
2	Trout	+	4.8	7
3	Trout	+	6.3	4
4	Trout	+	6.3	7
5	Char	+	4.8	4
6	Char	+	4.8	7
7	Char	+	6.3	4
8	Char	+	6.3	7
9	Trout	-	4.8	4
10	Trout	-	4.8	7
11	Trout	-	6.3	4
12	Trout	-	6.3	7
13	Char	-	4.8	4
14	Char	-	4.8	7
15	Char	-	6.3	4
16	Char	-	6.3	7
17 ^b^	Char	+	4.8	7

^a^ Three containers of each condition were prepared for the experiment. ^b^ P100 anti-*Listeria* phages were also added.

## Supplementary Materials

The following are available online at https://www.mdpi.com/2304-8158/9/2/119/s1: Figure S1: Growth of background flora, measured as total anaerobic counts (CFU/mL brine) in the different *rakfisk* batches. Data point for ‘Char, 4.8% NaCl, Day 14’ is missing. Supplementary File: Supplementary material to “Growth Behavior of *Listeria monocytogenes* in a Traditional Norwegian Fermented Fish Product (*Rakfisk*), and Its Inhibition through Bacteriophage Addition”.
