# Supplementary material for: Growth Behavior of Listeria monocytogenes in a Traditional Norwegian Fermented Fish Product (Rakfisk), and Its Inhibition through Bacteriophage Addition
Source: Foods. 2020 Jan 22;9(2):119. doi: 10.3390/foods9020119 (PMC7074442; doi:10.3390/foods9020119)
Supplement: Supplementary file 1 [file foods-09-00119-s001.pdf]

Supplementary material to:

"Growth Behavior of *Listeria monocytogenes* In a Traditional Norwegian Fermented Fish Product (*Rakfisk*), and its Inhibition Through Bacteriophage Addition."

by Axelsson et al.

Statistics

See also Materials and Methods, section 2.9, in main manuscript.

3.1 Chemical Measurements

ANOVA – Organic acids
model: y = mean + Species + Temp + Salt + Species*Temp + Species*Salt + Temp*Salt + Species*Temp*Salt + error.
Fitted using lm in R

```
Response: Lactic acid
                   Df Sum Sq Mean Sq F value   Pr(>F)
Temp                1  26.92  26.919  1.1540 0.290061
Salt                1  75.03  75.034  3.2 167 0.081535 .
Species             1 184.30 184.298  7.9008 0.008039 **
Temp:Salt           1   0.04   0.043  0.0018 0.966092
Temp:Species        1  36.01  36.013  1.5439 0.222300
Salt:Species        1  11.98  11.978  0.5135 0.478381
Temp:Salt:Species   1  33.34  33.343  1.4294 0.239900
Residuals          35 816.42  23.326
---
Response: Formic acid
                   Df Sum Sq Mean Sq  F value    Pr(>F)
Temp                1  58.46   58.46  11.7939 0.0015455 **
Salt                1 632.41  632.41 127.5860 3.188e-13 ***
Species             1  75.56   75.56  15.2440 0.0004113 ***
Temp:Salt           1  18.86   18.86   3.8051 0.0591398 .
Temp:Species        1   0.21    0.21   0.0417 0.8393945
Salt:Species        1   6.98    6.98   1.4077 0.2434330
Temp:Salt:Species   1  11.08   11.08   2.2344 0.1439355
Residuals          35 173.48    4.96
---
Response: Acetic acid
                   Df Sum Sq Mean Sq  F value    Pr(>F)
Temp                1 626.40  626.40 144.3972 5.631e-14 ***
Salt                1 995.60  995.60 229.5051 < 2.2e-16 ***
Species             1  35.76   35.76   8.2435  0.006898 **
Temp:Salt           1 273.69  273.69  63.0901 2.422e-09 ***
Temp:Species        1   1.89    1.89   0.4348  0.513949
Salt:Species        1  17.28   17.28   3.9825  0.053804 .
Temp:Salt:Species   1   8.58    8.58   1.9784  0.168380
Residuals          35 151.83    4.34
---
Signif. codes:  0 '***' 0.001 '**' 0.01 '*' 0.05 '.' 0.1 ' ' 1
```

3.2 Growth of L. monocytogenes in the Brine at Two Different Temperatures and Salt Concentrations During the Ripening Process of Rakfisk

ANOVA – Growth curves

Analysis of Variance Table. TROUT

```
Response: logCFU
          Df  Sum Sq Mean Sq F value    Pr(>F)    
Temp       1 26.0449 26.0449 99.7920 3.002e-16 ***
NaCl       1  0.0992  0.0992  0.3800   0.53914    
Day        1  4.9246  4.9246 18.8688 3.667e-05 ***
Temp:Day   1  9.2632  9.2632 35.4923 4.895e-08 ***
NaCl:Day   1  1.1049  1.1049  4.2334   0.04253 *  
Residuals 90 23.4893  0.2610                      
---
Signif. codes:  0 '***' 0.001 '**' 0.01 '*' 0.05 '.' 0.1 ' ' 1
```

Analysis of Variance Table. Char

```
Response: logCFU
              Df  Sum Sq Mean Sq F value    Pr(>F)    
Temp           1 24.8678 24.8678 70.0949 8.018e-13 ***
NaCl           1  2.5803  2.5803  7.2731 0.0083876 ** 
Day            1  0.0034  0.0034  0.0095 0.9227162    
Temp:NaCl      1  0.1586  0.1586  0.4471 0.5054524    
Temp:Day       1  5.1814  5.1814 14.6048 0.0002468 ***
NaCl:Day       1  0.3066  0.3066  0.8642 0.3551195    
Temp:NaCl:Day  1  2.4565  2.4565  6.9241 0.0100404 *  
Residuals     88 31.2201  0.3548                      
---
Signif. codes:  0 '***' 0.001 '**' 0.01 '*' 0.05 '.' 0.1 ' ' 1
```

ANOVA – Specific days

```
Dag 14: Analysis of Variance Table
Response: logCFU
               Df Sum Sq Mean Sq F value    Pr(>F)
Temp            1 8.8950  8.8950 71.2559 2.744e-07 ***
NaCl            1 3.1974  3.1974 25.6133 0.0001158 ***
Fish            1 1.3085  1.3085 10.4823 0.0051534 **
Temp:NaCl       1 0.7191  0.7191  5.7604 0.0289145 *
Temp:Fish       1 0.1057  0.1057  0.8466 0.3711801
NaCl:Fish       1 0.0066  0.0066  0.0525 0.8216645
Temp:NaCl:Fish  1 0.0161  0.0161  0.1288 0.7243823
Residuals      16 1.9973  0.1248
---
Signif. codes:  0 '***' 0.001 '**' 0.01 '*' 0.05 '.' 0.1 ' ' 1
```

```
Dag 28: Analysis of Variance Table
Response: logCFU
               Df  Sum Sq Mean Sq F value    Pr(>F)
Temp            1 15.3432 15.3432 46.3450 4.208e-06 ***
NaCl            1  0.4664  0.4664  1.4087    0.2526
Fish            1  0.3400  0.3400  1.0271    0.3259
Temp:NaCl       1  0.7749  0.7749  2.3407    0.1456
Temp:Fish       1  0.0866  0.0866  0.2614    0.6161
NaCl:Fish       1  0.1044  0.1044  0.3155    0.5821
Temp:NaCl:Fish  1  0.0007  0.0007  0.0022    0.9628
Residuals      16  5.2970  0.3311
---
Signif. codes:  0 '***' 0.001 '**' 0.01 '*' 0.05 '.' 0.1 ' ' 1
```

```
Dag 91: Analysis of Variance Table
Response: logCFU
               Df  Sum Sq Mean Sq F value    Pr(>F)
Temp            1 14.9906 14.9906 62.8924 6.209e-07 ***
NaCl            1  0.0784  0.0784  0.3290   0.57424
Fish            1  0.1207  0.1207  0.5064   0.48697
Temp:NaCl       1  0.2343  0.2343  0.9832   0.33618
Temp:Fish       1  0.0123  0.0123  0.0516   0.82322
NaCl:Fish       1  0.4578  0.4578  1.9205   0.18482
Temp:NaCl:Fish  1  0.7712  0.7712  3.2354   0.09095 .
Residuals      16  3.8137  0.2384
---
Signif. codes:  0 '***' 0.001 '**' 0.01 '*' 0.05 '.' 0.1 ' ' 1
```

3.3 Effect of Phage P100 on L. monocytogenes *During the Ripening of* Rakfisk

Phage experiment - Growth curve

```
Type III Analysis of Variance Table with Satterthwaite's method

               Sum Sq Mean Sq NumDF DenDF F value   Pr(>F)
Treatment      4.4389  4.4389     1    75  8.4001 0.004917 **
Day            4.2033  4.2033     1    75  7.9544 0.006134 **
Treatment:Day 0.1049  0.1049     1    75  0.1985 0.657247
---
Signif. codes:  0 '***' 0.001 '**' 0.01 '*' 0.05 '.' 0.1 ' ' 1
```

3.4 Effect of Phage P100 Addition on L. monocytogenes in Ripened Rakfisk Products During Short Term Storage

```
Analysis of Variance Table

Response: log cfu/g

               Df Sum Sq Mean Sq F value  Pr(>F)
Treatment       2 2.3309 1.16543  3.1393 0.08006 .
Day             1 0.3532 0.35323  0.9515 0.34859
Treatment:Day  2 0.1554 0.07771  0.2093 0.81403
Residuals      12 4.4548 0.37123
---
Signif. codes:  0 '***' 0.001 '**' 0.01 '*' 0.05 '.' 0.1 ' ' 1
```

Supplementary Figure S1

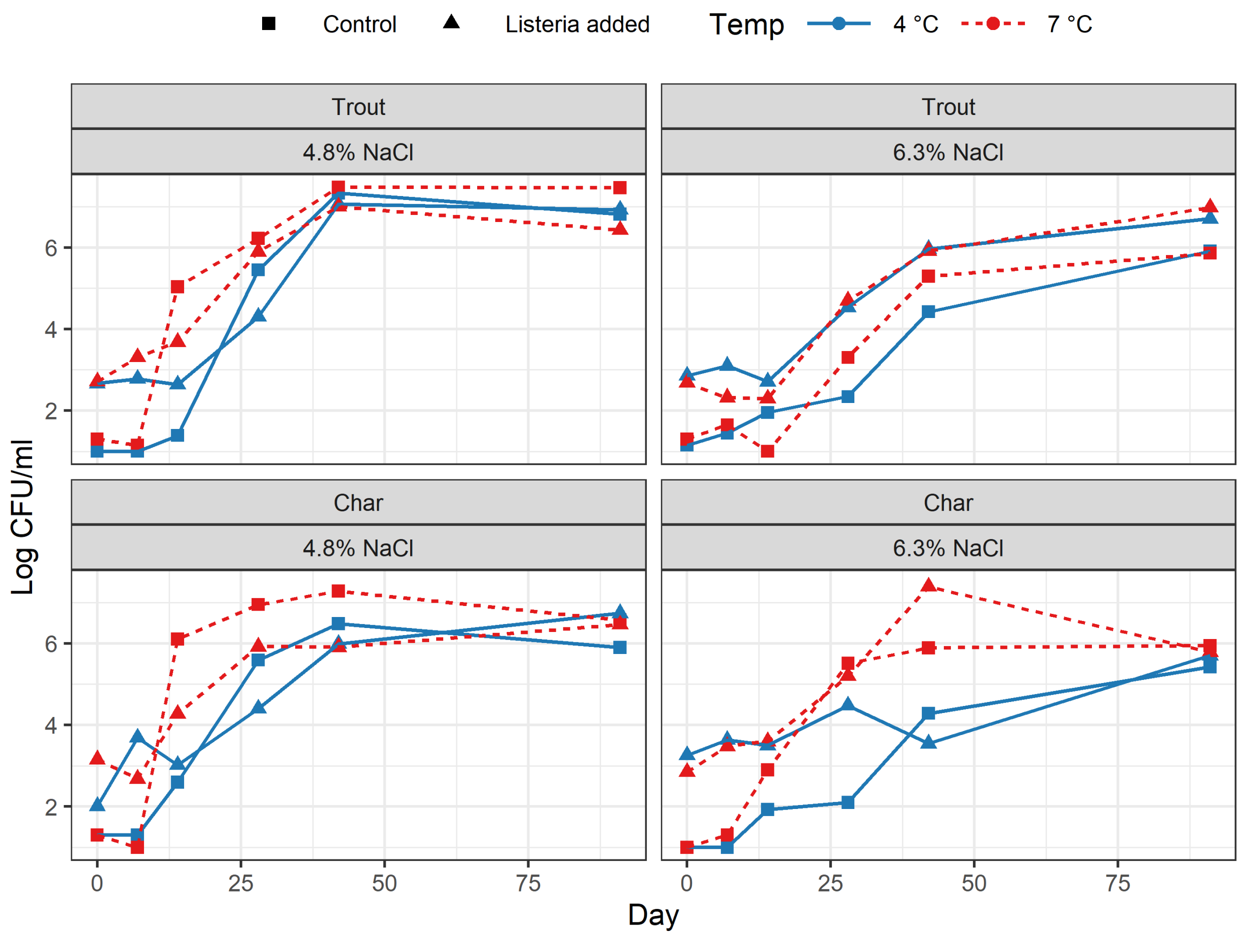

Figure S1: Growth of background flora, measured as total anaerobic counts (CFU/mL brine) in the different *rakfisk* batches. Data point for 'Char, 4.8% NaCl, Day 14' is missing.
